# Skin-to-skin contact—An effective intervention on pain and stress reduction in preterm infants

**DOI:** 10.3389/fped.2023.1148946

**Published:** 2023-03-22

**Authors:** Halyna Pavlyshyn, Iryna Sarapuk

**Affiliations:** Department of Pediatrics No 2, I. Horbachevsky Ternopil National Medical University, Ternopil, Ukraine

**Keywords:** preterm infants, pain, stress, skin-to-skin contact, laboratory markers

## Abstract

**Introduction:**

The outcomes of pain and stress in preterm infants in the neonatal intensive care units (NICUs) compel the continued search for pain- and stress-reducing interventions.

**The objective of the study:**

To investigate how skin-to-skin contact (SSC) influences chronic pain and stress in preterm infants in the NICU.

**Materials and methods:**

The study included 140 preterm infants in the NICU with gestational age less than 34 weeks. The overall design was a baseline-response design. Urine and saliva were collected before (baseline) and after SSC to measure pain and stress markers by enzyme immunoassay method. The behavioral indicators of chronic pain were assessed using the EDIN (Échelle Douleur Inconfort Nouveau-Né—neonatal pain and discomfort).

**Results:**

There was a significant decrease in the dopamine level in preterm infants after SSC in comparison with baseline values (85.99 [69.35; 112.20] pg/ml vs. 132.20 [104.80; 183.70] pg/ml), *p* < 0.001. The β-endorphin and serotonin levels increased after SSC (40.09 [26.81; 70.63] pg/ml vs. 29.87 [20.61; 46.94] pg/ml, *p* = 0.009 and 25.49 [20.45; 40.08] ng/ml vs. 22.30 [15.13; 31.65] ng/ml, *p* = 0.011, respectively). A significant decrease in cortisol levels in saliva and urine after SSC in comparison with baseline values (0.125 [0.079; 0.225] μg/dl vs. 0.371 [0.188; 1.002] μg/dl, *p* = 0.000 and 27.06 [14.59; 35.35] ng/ml vs. 35.25 [19.78; 61.94] ng/ml, *p* = 0.001, with a simultaneous increase of oxytocin level (57.00 [36.55; 88.49] pg/ml vs. 38.20 [28.78; 56.04] pg/ml, *p* = 0.009 were revealed. The total pain EDIN score in infants after SSC was below 6 points, significantly decreasing compared to the baseline (*p* < 0.05).

**Conclusion:**

Preterm infants in the NICU experience stress and pain, which were confirmed by the EDIN pain scale and laboratory markers. The level of dopamine and cortisol as pain and stress hormones were reliably high, and normalized after regular SSC. Simultaneously, pain-relieving and anti-stress markers of oxytocin, β-endorphin and serotonin reliably increased in preterm infants in response to the SSC.

## Introduction

Advances in neonatology have significantly reduced morbidity and mortality, but neonatal pain, discomfort, and stress remain significant problems in neonatal intensive care units (NICUs), especially for preterm infants (1). Their first days and sometimes months of life are spent in an overstimulating environment with many painful and harmful procedures, physically separated from their mothers (2, 3). Since Anand et al. published data showing that nociceptive activity constitutes a huge physiological stress for infants (4), there has been significant progress in the study of neonatal pain. However, in some neonatal intensive care units, most painful procedures are performed without anesthesia (5). But, newborns experience pain and need the same quality of pain assessment and treatment as adults (6). According to the International Association for the Study of Pain, the inability to communicate verbally does not eliminate the possibility that the child is in pain and needs appropriate pain-relieving treatment (7).

Newborn infants in the intensive care unit are exposed to numerous painful procedures. Hospitalized neonates were reported to undergo 10–18 painful procedures per day during their NICU stay (8, 9). A recent multicenter prospective observational study EPIPPAIN 2 has also shown that newborns have an average of 16 heel sticks and 4 venipunctures during an 8-day intensive care unit stay without routine analgesics (10). A total of 74% of the 3,000 registered procedures are performed on extremely and very preterm infants (11, 12). Cong et al. showed that preterm infants experienced a total of 643 acute painful procedures (23 daily) and 1,193 h of chronic events (43 h daily) cumulatively) during the first 4 weeks of intensive care unit stay (13).

The issue of chronic pain assessment and treatment remains no less relevant. A recent international prospective observational study examining neonatal pain assessment practices in 243 European intensive care units in 18 countries found that acute pain was assessed every 4–6 h, but only 10% of neonates received a daily assessment of permanent chronic pain (14), and there are no data on its management.

Despite the fact that the brain of preterm infants is in a critical period of development, they have both anatomical and neurochemical pain perception capabilities, and their ascending pain pathways are functional from 24 weeks of gestation (5, 15). Tactile perception threshold is lower and descending inhibitory pathways are immature in preterm infants, thus they are very sensitive to repeated and prolonged pain and have a more pronounced reflex response even to touch (7, 13). Touch stimulation near the injured area causes intense pain for several days or weeks (5). Due to imbalanced levels of afferent pain neurotransmitters, which are abundant at birth, and neurotransmitters of descending inhibitory pathways, preterm infants have a limited ability to modulate the pain (16).

Uncontrolled chronic pain in newborns leads to significant short-term and long-term adverse outcomes. The short-term consequences of painful procedures are manifested by the vital signs disturbances (heart rate variability, desaturation, apnea, arterial hypertension, intracranial pressure fluctuations) (17, 18), leading to the depletion of insufficient energy reserves in a preterm child, increasing the risk of morbidity and mortality (5). Chronic pain can affect growth, immune function, recovery, and length of hospital stay (1).

Experiences of chronic pain and stress early in life, together with repeated painful procedures, during a critical period of neurological development are associated with significant long-term neurological morbidity (13, 19), in particular, with lower cognitive and motor development (20, 21). Early life pain experiences have the ability to influence pain modulation and pain response in adulthood (15), lead to long-term local and diffuse disturbances in pain perception (22) and contribute to chronic pain syndrome formation, sleep and mood disorders (5, 15). Therefore, pain management in preterm infants is necessary not only for ethical reasons, but also for the purpose of reducing pain-related complications and preventing pain-induced negative impacts on the child's development.

Although significant progress has been made in the treatment and prophylaxis of neonatal pain in many intensive care units, the early and long-term outcomes of pain and stress in these vulnerable infants compel the continued search for healing pain- and stress-reducing interventions. It has long been known that close contact between mother and newborn infant is the natural way to reduce stress for both (23). Kangaroo care has a variety of benefits for infants and parents, including improved physiological stability, reduced risk of nosocomial infection, early weight gain, longer periods of quiet sleep, improved self-regulation, neurodevelopment and breastfeeding, reduced stress and decreased pain perception, enhanced parent-infant attachment, increased parental self-confidence, greater parental ability to recognize their infant's cues, decreased parental stress and postnatal depression (24–27).

### The objective of the study

To investigate how skin-to-skin contact influences chronic pain and stress intensity in preterm infants in the NICU by assessing laboratory markers.

## Materials and methods

The study included 140 preterm infants with gestational age less than 34 weeks, who were treated in the level III NICU of the regional perinatal center. Prematurity [gestational age (GA) ≤ 33 + 6/7 weeks] was the criteria for inclusion in this study. Exclusion criteria were the following: congenital malformations, chromosomal diseases, and absence of parents' consent.

The intensity of chronic pain in preterm infants was assessed using the EDIN scale (Échelle Douleur Inconfort Nouveau-Né—neonatal pain and discomfort scale), which includes 5 behavioral indicators of chronic pain: facial activity, body movements, sleep quality, quality of contact with medical staff, and consolability. Each indicator was scored from 0 to 3. The total EDIN score >6 indicated intensive chronic pain. For the laboratory evaluation of pain and the effectiveness of SSC in pain and stress relief, the level of markers of pain (dopamine, serotonin, endorphin in urine) and stress (cortisol in saliva and oxytocin in urine) were determined before (baseline level) and after skin-to-skin contact of an infant with mother.

For the laboratory evaluation of pain and the effectiveness of skin-to-skin contact (SSC) in pain and stress relief the level of pain markers (dopamine, serotonin, endorphin in urine) and stress markers (urinary and salivary cortisol and urinary oxytocin) were determined before (baseline level) and after rooming-in and skin-to-skin contact of an infant with mother. The overall design of the study was a baseline-response design. The baseline was defined as the state before the SSC was introduced. The response refers to the pain and stress response in relation to the SSC introduced. SSC lasted a minimum of 60 min and more, and the examination was performed after the SSC cycle (at least after 2 days of kangarooing). The intervention was provided on the 4th–10th day of the infant's life. Baseline samples were collected one hour prior to SSC and then saliva and urine samples were collected immediately after and two hours after SSC, respectively.

All infants included in the study had the SSC with their mothers. Naked preterm newborns were placed on the mother's chest dressed only in a hat and nappy so that the frontal contact of the mother and baby was skin to skin.

### Sample collection and hormones (dopamine, β-endorphin, serotonin, cortisol and oxytocin) assay

Saliva and urine were collected before (baseline) and after skin-to-skin contact. Urine and saliva samples were collected using cotton sponges after that was extracted from the sponges by centrifugation (2 min at 2,000× g). Saliva samples were collected without the usage of any salivation-stimulating agents. After extraction, saliva samples were frozen and stored at −20°C, urine samples were centrifuged for 20 min at 1,000× g at 2–8°C and after that were frozen and stored at −80°C.

Enzyme immunoassay kits for the quantitative determination of dopamine (Dopamine Elisa kit, Elabscience, Wuhan, China), β-endorphin (β-endorphin Elisa kit, Elabscience, Wuhan, China), serotonin (Serotonin Elisa kit, Elabscience, Wuhan, China), cortisol (Urinary Cortisol ELISA, Diametra, Perugia, Italy), and oxytocin (Oxytocin Elisa kit, Elabscience, Wuhan, China) were used to analyze hormones concentrations in the urine samples. Enzyme immunoassay kit for the quantitative determination of free cortisol in human saliva was used to analyze cortisol concentrations in the saliva samples (IBL International GmbH, Hamburg, Germany). Samples were analyzed in duplicate, and assays were performed using provided controls according to the manufacturer's instructions.

### Human research statement

Ethics approval was obtained from the appropriate local ethics committee and research was conducted under the World Medical Association's Helsinki Declaration. Informed consent was obtained from all parents whose infants took part in the study.

### Statistics

All computations were performed using StatSoft STATISTICA Version 13 (Tulsa, OK, United States). Quantitative data are presented as the median and interquartile range (IQR; 25th to 75th percentiles). For qualitative parameters, absolute and relative frequencies are presented. Wilcoxon matched pairs test (for two dependent groups) was used to identify differences in pre-post levels of laboratory markers. Kruskal–Wallis test (for multiple independent groups) was used to identify differences in pain and stress markers in preterm infants of different GA groups. Significance was assumed at *p* < 0.05 level. The required sample size was calculated using G*Power Software sample size calculator.

## Results

There were 140 preterm infants, including 19 extremely preterm (13.6%), 52 very preterm (51.4%), and 49 moderate preterm infants (35%). There were 74 (52.9%) males and 66 (47.1%) females; 54 twins (38.6%) and 86 singletons (61.4%). The mean gestational age (GA) of the study population was (31.1 ± 2.4) weeks, the mean birth weight—(1,591.46 ± 439.51) grams, the mean length at birth—(39.96 ± 4.25) cm, the mean head circumference—(28.92 ± 2.37) cm. Fourteen infants (10.0%) were born small for gestational age. The history of pregnancy and delivery, and anthropometric indicators of the study population depending on the GA are presented in Table 1. The clinical characteristics of the study population depending on the GA are presented in Table 2.

**Table 1:** History of pregnancy and delivery, anthropometric indicators of the study population.

	Statistical indicator	Extremely preterm infants, *n* = 19	Very preterm infants, *n* = 72	Moderate preterm infants, *n* = 49	*p* for Pearson's *χ*^2^ test
Singleton pregnancy	*n* (%)	15 (78.95)	42 (58.33)	29 (59.18)	*χ*^2 ^= 2.86; *р *= 0.239
Multiple pregnancies	*n* (%)	4 (21.05)	30 (41.67)	20 (40.82)	
Type of delivery					
vaginal	*n* (%)	7 (36.84)	20 (27.78)	12 (24.49)	*χ*^2 ^= 1.04; *р *= 0.594
cesarean section	*n* (%)	12 (63.16)	52 (72.22)	37 (75.51)	
Anthropometric indicators					
Birth weight, grams	Mean ± SD	917.37 ± 207.14	1,559.72 ± 281.42	1,899.49 ± 384.58	*p*_1–2 _= 0.0000*
					*p*_1–3 _= 0.0000*
					*p*_2–3 _= 0.0000*
Birth weight, percentile	Mean ± SD	53.68 ± 27.93	59.17 ± 25.43	44.31 ± 29.51	*p*_1–2 _= 0.715
					*p*_1–3 _= 0.410
					*p*_2–3 _= 0.009*

*Statistically significant results.

**Table 2:** Clinical characteristics of the of study population.

	Statistical indicator	Extremely preterm infants, *n* = 19	Very preterm infants, *n* = 72	Moderate preterm infants, *n* = 49	*p* for Pearson's *χ*^2^ test
Apgar score in the 5th min < 7	*n* (%)	10 (52.63)	11 (15.28)	0	*χ*^2 ^= 29.75; *р* = 0.000*
Neonatal resuscitation	*n* (%)	18 (94.74)	41 (56.94)	13 (26.53)	*χ*^2 ^= 27.30; *р* = 0.000*
Surfactant replacement therapy	*n* (%)	18 (94.74)	24 (35.29)	1 (2.08)	*χ*^2 ^= 54.58; *р* = 0.000*
Respiratory distress syndrome	*n* (%)	19 (100.00)	60 (83.33)	21 (42.86)	*χ*^2 ^= 32.20; *р* = 0.000*
Early-onset neonatal sepsis	*n* (%)	8 (42.11)	13 (18.06)	12 (24.49)	*χ*^2 ^= 4.86; *р* = 0.087
Hypoxic-ischemic encephalopathy (moderate + severe)	*n* (%)	11 (57.89)	11 (15.28)	9 (18.37)	*χ*^2 ^= 16.46; *р* = 0.0003*
Intraventricular hemorrhage (I + II stages)	*n* (%)	9 (47.37)	9 (12.50)	12 (24.49)	*χ*^2 ^= 11.27; *р* = 0.0036*
Intraventricular hemorrhage (III–IV stages)	*n* (%)	2 (10.53)	1 (1.39)	0	*χ*^2 ^= 7.64; *р* = 0.022*
Neonatal seizures	*n* (%)	9 (47.37)	9 (12.50)	8 (16.33)	*χ*^2 ^= 12.34; *р* = 0.002*
Late-onset neonatal sepsis	*n* (%)	11 (57.89)	10 (13.89)	4 (8.16)	*χ*^2 ^= 24.68; *р* = 0.000*
Necrotizing enterocolitis	*n* (%)	2 (10.53)	17 (23.61)	10 (20.41)	*χ*^2 ^= 1.57; *р* = 0.456
Need for mechanical ventilation	*n* (%)	15 (78.95)	19 (26.39)	11 (22.45)	*χ*^2 ^= 22.28; *р* = 0.000*

*Statistically significant results.

Clinical assessment of pain in preterm infants revealed an EDIN score above 6 points in 55.5% of the children, which indicated intense chronic pain in the intensive care unit. The median of the total pain index on the EDIN scale was 6.0 [5.0; 7.0] points. Disturbed facial activity and quality of contact with medical staff were the most frequent and intense signs of chronic pain. Significantly higher scores on the EDIN pain scale were found in extremely preterm infants [7.0 (7.0; 8.0) points] compared to very 6.0 [5.0; 6.5] points) and moderate preterm neonates [5.0 (4.0; 6.0) points], *H* = 13.24; *p* = 0.001. The total pain score of all infants during and after SSC was below 6 points, significantly decreasing compared to the baseline (*p* < 0.05).

The laboratory investigation of pain and stress in preterm infants in the NICU was conducted using markers (dopamine, serotonin, endorphin, cortisol, and oxytocin) associated with chronic pain and stress. Levels of laboratory pain and stress indices in preterm infants depending on GA are presented in Table 3.

**Table 3:** Laboratory markers of chronic pain and stress in preterm infants.

Marker	Extremely preterm infants, *n* = 19	Very preterm infants, *n* = 72	Moderate preterm infants, *n* = 49	Kruskal–Wallis test	*Р*
Dopamine in urine, pg/ml	136.10 (111.80; 163.90)	121.80 (91.43; 165.20)	159.55 (112.50; 238.90)	*H* = 3.44; *p* = 0.179	*p*_1–2 _= 1.000
					*p*_1–3 _= 1.000
					*p*_2–3 _= 0.216
β-endorphin in urine, pg/ml	26.48 (14.23; 44.60)	32.80 (21.99; 50.95)	23.80 (18.94; 37.81)	*H* = 2.06; *p* = 0.357	*p*_1–2 _= 1.000
					*p*_1–3 _= 1.000
					*p*_2–3 _= 0.594
Serotonin in urine, ng/ml	22.71 (14.77; 26.73)	24.03 (17.55; 32.08)	18.75 (14.26; 36.31)	*H* = 0.68; *p* = 0.712	*p*_1–2 _= 1.000
					*p*_1–3 _= 1.000
					*p*_2–3 _= 1.000
Cortisol in saliva, μg/dl	0.734 (0.187; 2.010)	0.325 (0.161; 0.801)	0.246 (0.111; 0.585)	*H* = 6.44; *p* = 0.040*	*p*_1–2 _= 0.142
					*p*_1–3 _= 0.033*
					*p*_2–3 _= 0.986
Cortisol in urine, ng/ml	48.35 (37.34; 109.50)	35.44 (18.97; 63.58)	26.68 (16.19; 42.26)	*H* = 7.26; *p* = 0.026*	*p*_1–−2 _= 0.094
					*p*_1–3 _= 0.021*
					*p*_2–3 _= 0.824
Oxytocin in urine, pg/ml	39.09 (30.55; 52.52)	39.96 (28.78; 62.50)	34.14 (22.35; 61.19)	*H* = 0.20; *p* = 0.904	*p*_1–2 _= 1.000
					*p*_1–3 _= 1.000
					*p*_2–3 _= 1.000

*Statistically significant results.

No association was found between the dopamine, β-endorphin, serotonin, and oxytocin levels in preterm infants and GA, however, cortisol levels in saliva and urine were significantly higher in extremely preterm infants compared to moderate preterms (Table 3). Negative correlations of cortisol level in saliva and urine and GA were revealed (*r* = −0.21; *p* = 0.029 and *r* = −0.23; *p* = 0.039, respectively).

**Figure 1:**
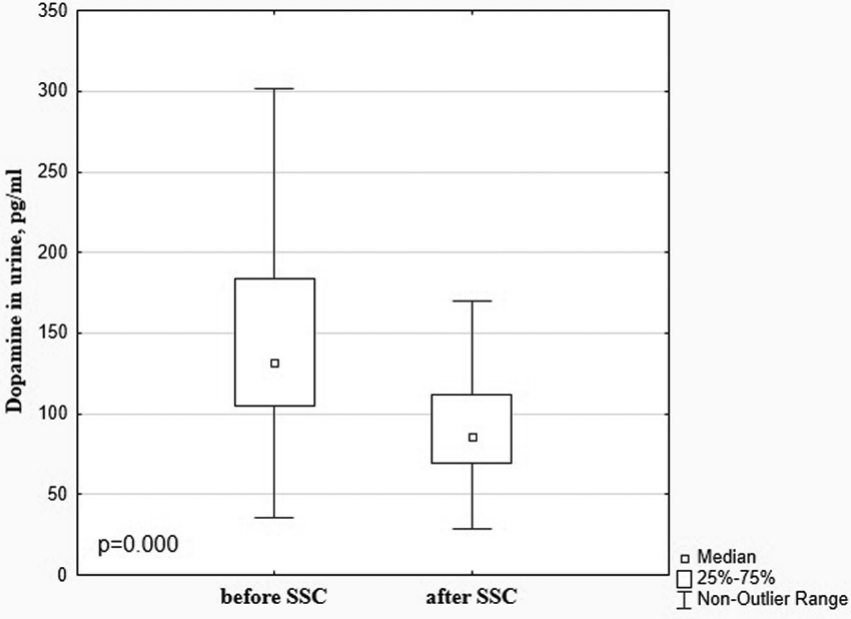
Dopamine in the urine in preterm infants before and after SSC.

**Figure 2:**
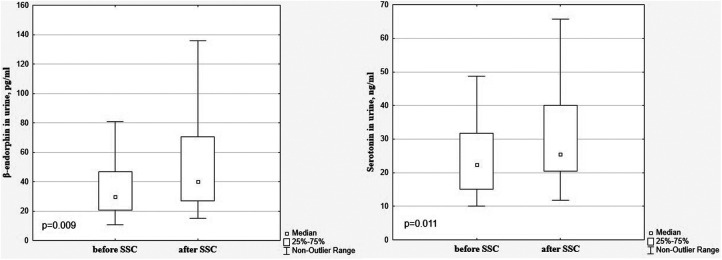
β-Endorphin and serotonin in the urine in preterm infants before and after SSC.

There was a significant decrease in the dopamine level in preterm infants after SSC in comparison with baseline values (85.99 [69.35; 112.20] pg/ml vs. 132.20 [104.80; 183.70] pg/ml), *p* < 0.001, Figure 1. The β-endorphin and serotonin levels simultaneously significantly increased after SSC compared to baseline values (40.09 [26.81; 70.63] pg/ml vs. 29.87 [20.61; 46.94] pg/ml, *p* = 0.009 and 25.49 [20.45; 40.08] ng/ml vs. 22.30 [15.13; 31.65] ng/ml, *p* = 0.011, respectively), Figure 2.

**Figure 3:**
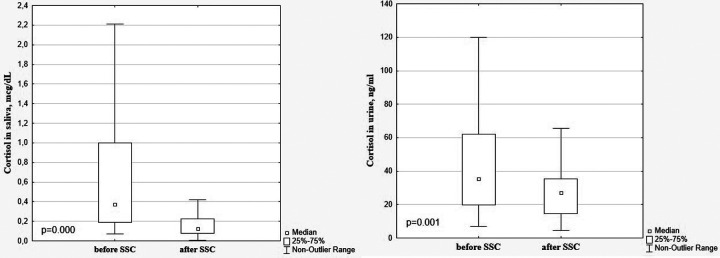
Cortisol in saliva and urine in preterm infants before and after SSC.

**Figure 4:**
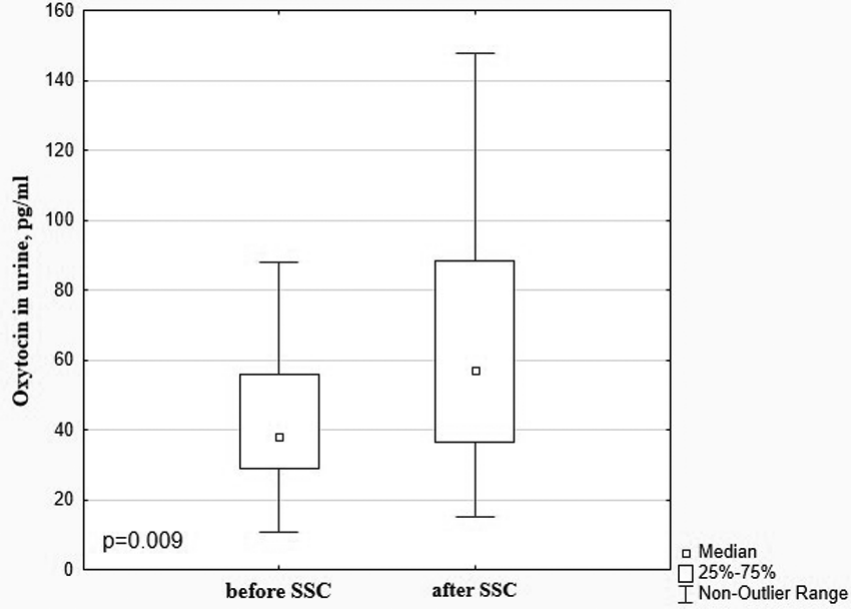
Oxytocin in the urine in preterm infants before and after SSC.

A significant decrease in cortisol levels in saliva and urine after SSC in comparison with baseline values (0.125 [0.079; 0.225] μg/dl vs. 0.371 [0.188; 1.002] μg/dl, *p* = 0.000 and 27.06 [14.59; 35.35] ng/ml vs. 35.25 [19.78; 61.94] ng/ml, *p* = 0.001, Figure 3) with a simultaneous increase of oxytocin level (57.00 [36.55; 88.49] pg/ml vs. 38.20 [28.78; 56.04] pg/ml, *p* = 0.009 (Figure 4) were revealed.

**Figure 5:**
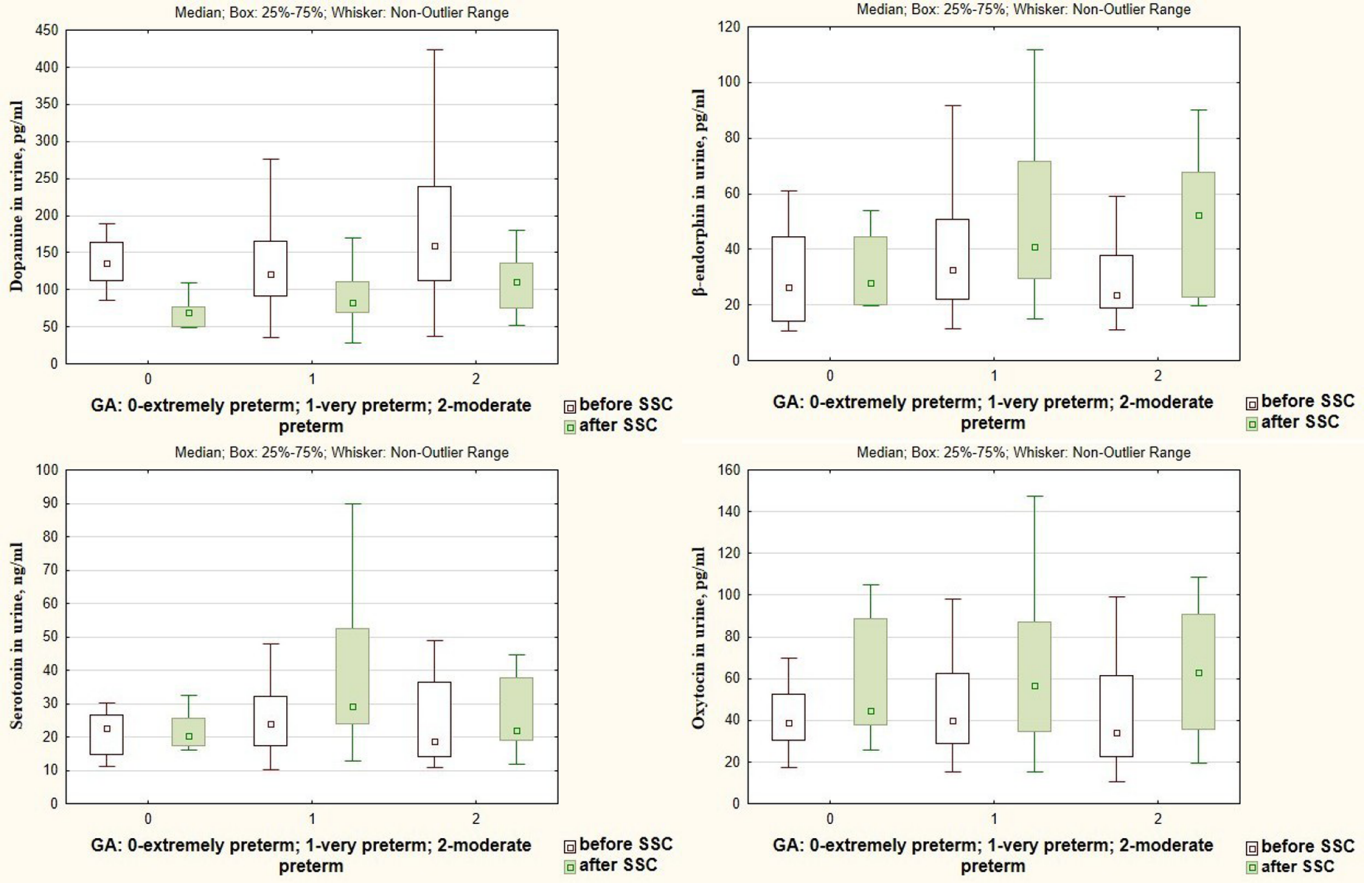
Dopamine, β-endorphin, serotonin, and oxytocin levels in urine before and after SSC in preterm infants of different gestational ages.

**Figure 6:**
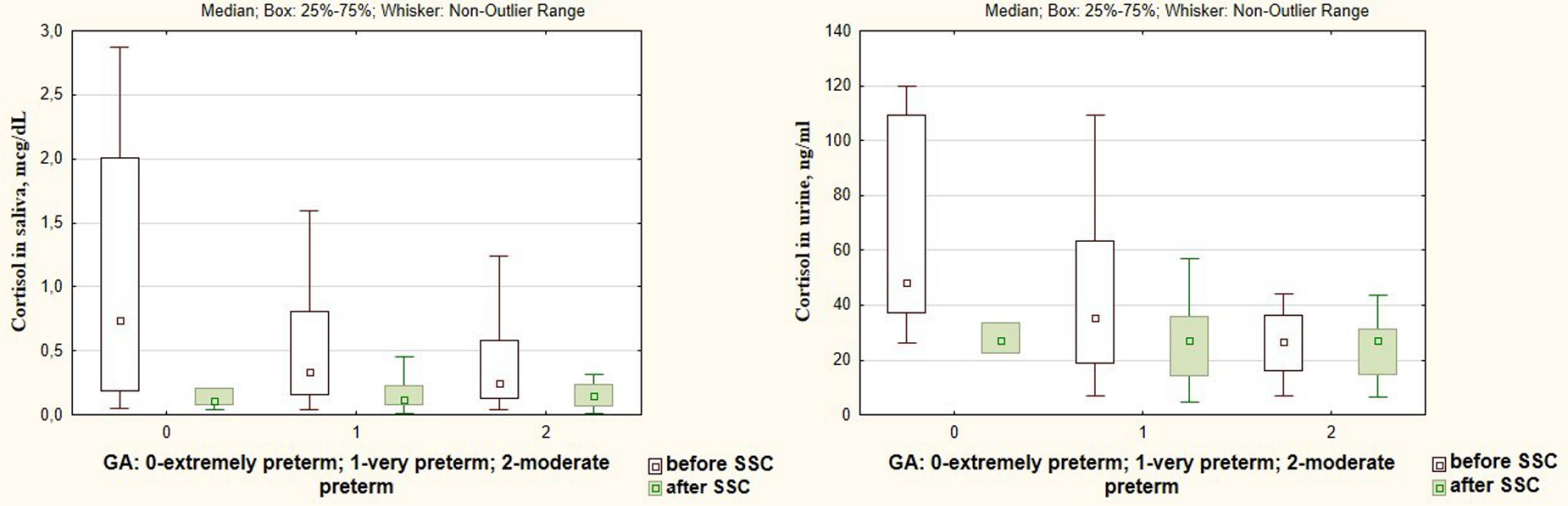
Cortisol levels in saliva and urine before and after SSC in preterm infants of different gestational age.

Changes in dopamine, β-endorphin, serotonin, and oxytocin levels in response to SSC were not associated with the gestational age of infants (*р* < 0.05), Figure 5. At the same time, there was found a more intense decrease in the salivary cortisol level in response to SSC in preterm infants with a smaller gestational age. Thus, salivary cortisol levels decreased by 10.3 times after SSC in extremely preterm newborns, by 5.5 times—in very preterm, and by 2.8 times in moderate preterm infants (*Н* = 6.77; *p* = 0.034), Figure 6.

Correlations between pain and stress markers were found. Thus, a reliable positive correlation between baseline dopamine and urinary cortisol levels was revealed (*r* = 0.49; *p* = 0.003). It was also found a positive correlation between baseline cortisol levels in urine and saliva (*r* = 0.38; *p* = 0.001). At the same time, there were reliable positive correlations between pain- and stress-relieving markers (β-endorphin, serotonin and oxytocin) both before and after SSC (Tables 4, 5).

**Table 4:** Correlation matrix of baseline β-endorphin, serotonin, and oxytocin levels.

	Baseline β-endorphine	Baseline serotonine	Baseline oxytocin
Baseline β-endorphine	Х		
Baseline serotonine	*r* = 0.73; *p* = 0.000	Х	
Baseline oxytocin	*r* = 0.75; *p* = 0.000	*r* = 0.57; *p* = 0.000	Х

**Table 5:** Correlation matrix of β-endorphin, serotonin, and oxytocin levels after SSC.

	β -endorphine after SSC	Serotonine after SSC	Oxytocin after SSC
β-endorphine after SSC	Х		
Serotonine after SSC	*r* = 0.65; *p* = 0.000	Х	
Oxytocin after SSC	*r* = 0.75; *p* = 0.000	*r* = 0.45; *p* = 0.005	Х

## Discussion

Our study is the first to determine the effect of SSC on chronic pain and stress laboratory markers. The results of our research shows the effectiveness of SSC as a non-pharmacological strategy to relieve pain and reduce stress in preterm infants during neonatal intensive care treatment.

A decrease in the dopamine level, a marker of the afferent pain perception system, and an increase in the β-endorphin and serotonin levels, markers of descending inhibitory pathways in response to the SSC indicates that this intervention helps in reducing the sensitivity to the perception of pain, as well as promotes pain modulation and decrease (28, 29). Preterm infants, especially extremely and very preterm, are exposed to repeated painful procedures during a period of intensive brain development and have well-developed nociceptive pathways for pain perception; however, their descending modulatory pain control is immature and lacks sufficient, so an imbalance between excitatory and inhibitory processes lead to increased nociceptive signaling in the central nervous system (1, 30, 31). The results of our study showed that preterm infants had high baseline dopamine and cortisol levels, which decreased after SSC, which indicates that these patients being treated in the NICU with numerous painful procedures and being separated from their parents in the early neonatal period, experienced pain and stress. High indices of the EDIN scale also confirmed this. Simultaneously, the baseline level of β-endorphin and serotonin, as pain modulators, was low compared to the after SSC values. This is consistent with and confirms the features of pain development in preterm infants. Namely, the afferent systems of pain perception, the marker of which is dopamine, are fully functional already from the 24th week of pregnancy; however, the descending inhibitory pathways, which markers are β-endorphin and serotonin, that modulate sensory experience, are immature (1, 32). Therefore, pain perception and stress response may be stronger in preterm infants (7). Although it was previously suspected that neonates have a suppressed and immature response to pain, it is now well established that full-term and preterm newborns have the neuroanatomical pathways necessary for nociception (7), and the results of our study confirm this with laboratory markers.

Other authors have also proven the effectiveness of SSC in reducing neonatal pain. However, all of these studies examined the effectiveness of the SSC on procedural pain modulation. Thus, Johnston et al., evaluating physiological and behavioral indicators of pain, proved that SSC is an effective intervention when performing a single painful procedure (12). Several researchers have found that SSC has a positive effect on the stabilization of vital signs (heart rate variability, oxygen saturation), duration of negative facial activity and crying, lower PIPP (premature infant pain profile) scores, and changes in brain activity measured by EEG monitoring during heel prick procedure in preterm infants (11, 33–35). Olsson et al. showed that SSC during venipuncture has an analgesic effect for preterm newborns measured with near-infrared spectroscopy (36).

It is also interesting to note that although holding a clothed baby provides some comfort, direct SSC is much more effective (37). But breastfeeding during the Kangaroo care enhances the healing analgesic effects of SSC (38), as well as feeding with expressed breast milk (39). Also, SSC is as effective or even more effective in relieving pain than glucose solutions (40, 41).

SSC has also been shown to improve an infant's sleep, increase the time without crying and reduce crying time (42), which is probably related to pain or stress. On the other hand, it is known that the state of restful sleep is associated with a reduced response to pain (43).

The analgesic effect of SSC is believed to be mediated by multisensory stimulation, neurochemical activation, and modulation of the stress regulatory system involved in the experience of pain (11). SSC provides continuous, non-phase, full-body touch, as well as parental warmth, heartbeat, body odor, voice, and interaction between parents and infants. All these components have soothing effects during invasive procedures. SSC activates the C-afferent fibers, and impulses from the stimulation of these nerves reach the insular cortex—the central part of the limbic region of the brain, thus activating the oxytocinergic system with the release of oxytocin (23, 44).

Oxytocin provides its anxiolytic and analgesic effect by shifting the autonomic nervous system from sympathetic to parasympathetic dominance (35, 45). Our study shows that cortisol levels decrease simultaneously with oxytocin levels increasing in the response to SSC. It is proven that oxytocin affects the hypothalamic-pituitary-adrenal axis by reducing cortisol levels (46, 47). Long-lasting and regular SSC, reducing stress in infants, also reduces the chronic pain sensation. Stress is thought to be an important factor in pain perception and pain response (48). Grunau et al., revealed that early and frequent pain sensation in preterm infants was associated with the development of a constant stress state (15). Positive correlations between dopamine and cortisol levels (*r* = 0.49; *p* = 0.003) confirm that pain and stress in preterm infants are interrelated processes. Jones et al. found much more intense pain response to routine heel prick procedures determined by EEG monitoring in infants who had already experienced NICU environment stress compared to those, who were not treated in the NICU (48). Kommers and colleagues found that oxytocin levels in response to SSC depended on the initial comfort status. They reported that infants who were more comfortable at baseline had increased oxytocin levels during SSC, whereas infants who were more uncomfortable at baseline had decreased oxytocin levels during SSC (49).

In addition, oxytocin increases the level of endorphins, which, in turn, reduces stress and pain effects (46). The positive correlations between oxytocin, β-endorphin, and serotonin before and after SSC revealed in our research, confirm the relationship of these neuroendocrine markers and coincide with the results of Henderson et al.

It is also known that SSC can elicit an innate tactile receptor response that regulates vagal tone and the release of endogenous opiates, oxytocin, and β-endorphins (43, 50), which was observed in our patients. There is also evidence that SSC can suppress nociceptive responses in the brain of preterm infants (51). Simple touch has been shown to modulate specific nociceptive responses in adults (52). Qiu et al. showed that combined music and touch intervention might decrease the pain response of preterm neonates by improving the β-endorphin concentration (53).

Changes in dopamine, β-endorphin, serotonin, and oxytocin levels in response to SSC were not associated with the gestational age of infants (*р* < 0.05), that indicated that SSC have been equally effective in all groups of preterm neonates. At the same time, a more intense decrease in the salivary cortisol level in response to SSC in extremely preterm infants compared to very and moderate preterm newborns indicate significant necessity of regular SSCs for the tiniest preterms.

It is quite difficult to differentiate between stress and pain in the neonatal period. It is believed that any painful sensation is stressful, but not every stress is painful (54). The stress-ameliorating effect of SSC has been studied by other authors. The results of our study are consistent with those of Vittner et al., who found that 60 min of SSC promote an increase in salivary oxytocin and a decrease in salivary cortisol in preterm infants (45). El-Farrash et al. studied the effectiveness of SSC for 60 and 120 min per day for 7 consecutive days in preterm infants compared with standard neonatal care and found that salivary cortisol levels were reduced much more significantly in the SSC groups (27).

The positive effects of parental presence in reducing pain and stress have also been shown in several studies (55, 56). Jones et al. found that maternal presence can reduce the cerebral cortex activity associated with harmful and excessive stimuli, and modulate the pathological neural processes caused by them (48). Thus, it is important to encourage the presence of parents in the NICU and promote family-centered care with regular SSC (10). Uvnas-Moberg et al. found that regular sessions of SSC produce a continuous stress-buffering anxiolytic effect for preterm infants in the NICU (23), while deprivation of a pleasant maternal touch can lead to toxic stress (57).

## Strengths and limitations

It is the first known research of SSC impact on laboratory mediators of chronic pain and stress in preterm infants. The correlation analysis between chronic pain and stress markers was evaluated for the first time. Recruitment into three different GA groups, from 24 to 34 weeks of gestation, was also a strength of this study. The main limitation of our study is the small size of the patient sample in the extremely preterm infants group.

## Conclusion

Preterm infants who were treated in the NICU with numerous painful procedures, and separated from their parents in the neonatal period experience stress and pain, which was confirmed by the EDIN pain scale and laboratory markers. Thus, the level of dopamine and cortisol as pain and stress hormones were reliably high, and normalized after regular SSC between the child and the mother. Simultaneously, pain-relieving and anti-stress markers of oxytocin, β-endorphin and serotonin reliably increased in preterm infants in response to the SSC.

## Data Availability

The raw data supporting the conclusions of this article will be made available by the authors, without undue reservation.
